# Quantifying volume and high-speed technical actions of professional soccer players using foot-mounted inertial measurement units

**DOI:** 10.1371/journal.pone.0263518

**Published:** 2022-02-03

**Authors:** Glyn Lewis, Christopher Towlson, Pietro Roversi, Chris Domogalla, Lee Herrington, Steve Barrett

**Affiliations:** 1 Performance and Medicine Department, Norwich City F.C., Norwich, United Kingdom; 2 Sport Science Department, University of Salford, Norwich, United Kingdom; 3 Department of Sport, Health and Exercise Science, University of Hull, Kingston upon Hull, United Kingdom; 4 Sport Science, Performance Analysis, Research and Coaching (SPARC), PlayerMaker, London, United Kingdom; Universidade de Evora, PORTUGAL

## Abstract

**Aims:**

The aims of the study were two-fold: i) examine the validity and reliability of high-speed kicking actions using foot-mounted inertial measurement unit’s (IMU), ii) quantify soccer players within-microcycle and inter-positional differences in both the frequency and speed of technical actions.

**Methods:**

During the in-season phase (25 weeks) of the UK domestic season, 21 professional soccer player ball releases, high-speed ball releases and ball release index were analysed. Pearson product-moment correlation coefficient and confidence intervals were used to determine the validity between the systems, whilst a general linear mixed model analysis approach was used to establish estimated marginal mean values for total ball releases, high-speed ball releases and ball release index.

**Results:**

Good concurrent validity was observed for ball release velocity and high-speed kicks against a high-speed camera (r^2^- 0.96, CI 0.93–0.98). Ball releases, high-speed ball releases and ball release index all showed main effects for fixture proximity (p>0.001), playing positions (p>0.001) and across different training categories (p>0.001). The greatest high-speed ball releases were observed on a match-day (MD)+1 (17.6 ± 11.9; CI- 16.2 to 19) and MD-2 (16.8 ± 15; CI- 14.9 to 18.7), with MD+1 exhibiting the highest number of ball releases (161.1 ± 51.2; CI- 155.0 to 167.2) and ball release index (145.5 ± 45.2; CI- 140.1 to 150.9) across all fixture proximities. Possessions (0.3 ± 0.9; CI- 0.3 to 0.4) and small-sided games (1.4 ± 1.6; CI- 1.4 to 1.5), had the lowest values for high-speed ball releases with technical (6.1 ± 7.2; CI- 5.7 to 6.6) and tactical (10.0 ± 10.5; CI- 6.9 to 13.1) drills showing the largest high-speed ball releases.

**Conclusions:**

The present study provides novel information regarding the quantification of technical actions of professional soccer players. Insights into absolute and relative frequency and intensity of releases in different drill types, provide practitioners with valuable information on technical outputs that can be manipulated during the process of planning training programmes to produce desired outcomes. Both volume and speed of ball release actions should be measured, when monitoring the technical actions in training according to fixture proximity, drill type and player position to permit enhanced training prescription.

## Introduction

Professional soccer training programmes prescribed by coaches are multi-factorial due to the physical, technical, tactical, and psychological demands of match-play [1]. Head coaches have been shown to favour technical and tactical-based soccer drills within tactical periodisation models of training philosophy, likely because of their direct relationship between technical and tactical-based outcomes on match-play success [2, 3]. While the physical outcomes of soccer training have been well-reported through time-motion analyses collected using global positioning systems (GPS; [1, 2, 4, 5], limited data is available exploring the volume and speed of technical actions (kicking actions defined as passes, crosses and shots) performed by soccer players [6, 7]. The primary objective of monitoring of external training loads is to maximise both performance and player availability, thus it has been suggested that this process should encompass all movements that require either a physiological or biomechanical stress to the athlete, including different mechanical actions such as kicking [8, 9]. Despite this, understanding the implications of performing an increased number of technical actions performed at higher speed in soccer and whether they should be incorporated into training load models requires further exploration. Further understanding of both the physical and technical outputs within professional soccer training and match-play may permit practitioners to prescribe more appropriate training programmes which are specific to the number of days pre-ceding/post-game day, along with identifying risk of injury during kicking actions [10] and planning return to play protocols specific to soccer players [11].

Monitoring technical actions within soccer training has been limited due to the time-consuming nature of manual coding of technical and tactical actions using traditional, video-based notation analysis methods [12]. Accompanied by the added consideration of human error or subjective identification of ball touches observed during live and retrospective video analysis [13]. To address this, Marris, Barrett [7] utilised foot-mounted inertial measurement units (IMUs) which were established to be a valid and reliable method for measuring players frequency of closed-skill technical actions. Findings here contrasted with typical physical micro-cycle tapering [4], with the number of performed sessional technical actions (i.e. ball touches and releases) being higher during a match-day minus one (i.e. MD-1), with training session and some training drill categories perhaps being mediated by positional specificity of the technical actions [7]. However, within this study, technical actions were limited to only absolute and relative data (per minute) across all positions, with no data presented on the actual speed of the technical actions. Furthermore, the speed of technical actions has been associated with the key performance outputs of soccer from a physical perspective [14], with minimal attention to implications of technical actions on the player. For example, Torreblanca-Martinez, Nevado-Garrosa [15] suggested that a players’ kicking velocity may have implications for their chance of gaining greater success during key attacking match activities such as shooting or crossing (considering accuracy). Kicking velocity has also been suggested to be a meaningful metric for quantifying a players’ workload during a training week and as part of a return to play monitoring tool [15, 16]. However, despite recent work reporting the frequency of technical actions reported using IMUs [7], the speed of technical actions is limited within the soccer training environment. Therefore, the aims of the current study were two-fold: i) examine the validity and reliability of high-speed kicking actions using foot-mounted IMU’s, ii) quantify soccer players within-microcycle, inter-positional differences in both the frequency and speed of technical actions.

## Methods

### Validity and reliability

Having ethical approval by the University of Salford Ethics Committee (HST1920-196) and all participants having provided informed written consent, the validity and reliability of the IMU equipment was established prior to the experimental element of the study. This was accomplished by measuring the velocity of the foot (equipped with an IMU) during a kicking action through statistical comparison with a criterion measure high-speed camera system (HSCS) analysis of joint angular velocity (Quintic Consultancy Ltd, Sutton Coldfield, UK). Which has been shown to be an appropriate method to assess high velocity actions [15]. Four male professional soccer players (central defenders (n = 1), wide defenders (n = 1), centre midfielder (n = 1), forward (n = 1); aged 28.1 ± 4.1 years, stature 184.5 ± 5.8cm, body-mass 78.7 ± 3.4kg) wore the foot-mounted IMU and were filmed simultaneously by HSCS at a captured rate of 240 frames per second. Players were given a fifteen-minute familiarisation session, during which they were instructed to kick the ball at low, moderate or high subjective intensities. Upon completion of the familiarisation, each player was required to execute 12 kicks of a static ball, with the surface of the foot they felt most comfortable with performing the kicking action at that speed (6 with each foot). This allowed for general comparison of systems across a range of subjective velocities and therefore, ball release intensities. Pearson product-moment correlation coefficient and 95% confidence intervals were used to determine the validity between the systems, with assessments made against small, moderate, large, and very large thresholds (± 0.1, 0.3, 0.5 and 0.7, respectively [17, 18]). Between system comparisons showed very large correlations across all velocities for recording ball release velocity, (r^2^- 0.96, CI 0.93–0.98). Within-session reliability of foot-mounted IMU was observed through analysis of the CV% at each subjective intensity of kick. Moderate to high reliability was observed across the intensities (14.35%, 5.82%, and 3.93% for low, moderate, and high ball release velocities, respectively).

### Experimental design

Having established the reliability and validity of the IMU`s, the technical actions of professional soccer players were quantified during training sessions throughout a twenty-five-week (September to March) mid-season period of the 2020/2021 English Football League Championship season. This phase ensured minimal changes to players’ physiological fitness, such as observed during the transition from pre-season to in-season, where coaches emphasise the continuation of physical conditioning [4, 7]. Two microcycles were excluded as they fell within the Fédération Internationale de Football Association International (FIFA) international match calendar [4, 19]. Training sessions within a microcycle were categorised in relation to the number of days prior to a competitive fixture (i.e., match-day (MD) minus day number [MD—#]) [4, 7]. Microcycles encompassing one fixture (n = %) typically contained four training sessions, with MD– 5/+2 being a recovery day for all players. Two-game microcycles contained 2–3 training sessions. Fixtures were followed by a recovery day for all players. According to their primary objective, training drills were assigned one of the following categories: position specific; possession; small-sided games (SSG); tactical; technical; or warm-up [2].

#### Exclusion criteria

Data was only included from players completing a minimum of three, full first team training sessions per fixture proximity following similar exclusion criteria set out in previous monitoring of technical actions in soccer [7]. A total of 105 group training sessions were included in the data set for analysis, which encompassed 1654 individual player observations. Data was analysed in relation to the number of days away from the competitive match day (MD-4, n = 10; MD-3, n = 12; MD-2, n = 13; MD-1, n = 39; MD+1, n = 31) in order to provide establish players technical and physical exposures to kicking within each of these sessions. Goalkeepers were not included in the study as it was considered that their training sessions were considered to be unique to their playing position [7]. Additional drills outside of the group training sessions were removed from the analysis. Any individual training or training as part of a return to play session, were also removed. Observations belonging to players who ceased training due to injury or load management purposes (n = 17; 1.0%) were removed from the analysis. Observations that contained missing data due to technological error (n = 33; 2.0%) were removed from analysis, leaving a total of 1604 observations for analysis.

Players completed a mean of 51.7 ± 26.3 training sessions, with 7.4 ± 2.1 drill observations per session. Recovery periods between drills were removed to provide an accurate representation of relative training outputs [20]. Each player completed 351.8 ± 98.1 drills during the study, which did not influence the training content delivered.

#### Participants

Twenty-one professional soccer players (mean ± SD age: 25.5 ± 4.6 years; stature: 182.8 ± 6.8 cm; body-mass: 78.8 ± 7.4 kg), from an English Football League Championship club (League Champions), participated in this study. The sample size was constrained by the finite number of players with professional contracts, who were available to participate in training, that satisfied the exclusion criteria [7]. As categorised by the head coach, who typically employed a 4-2-3-1 formation, the sample of players comprised of five central defenders (CD), five wide defenders (WD), six central midfielders (CM), three wide midfielders (WM) and two strikers (ST). The head coach and coaching staff remained consistent throughout, alleviating the potential influence of a change in head coach on the technical requirements of the training programme [2, 21].

#### Inertial measurement units

Ball releasing actions were quantified using commercially available foot-mounted IMUs (PlayerMaker™, Tel Aviv, Israel). Each IMU incorporated two components from the MPU-9150 multi-chip motion tracking module (InvenSense, California, USA), being a 16 g triaxial accelerometer and a 2000°•sec^-1^ triaxial gyroscope. Housed in manufacturer-supplied tightly fitting silicone straps, each player was equipped with two IMUs (one for each foot), which were located at the lateral malleoli over the player’s boots. To diminish issues related to inter-unit reliability, players used the same IMUs throughout the data collection period [22, 23]. Metrics derived from the foot-mounted IMU included, ball releases (total, dominant leg, non-dominant leg), high-speed ball releases (> 15m/s) and ball release index (total, dominant leg, non-dominant leg). Both absolute and relative outputs were established. Ball release index is a calculation that incorporates both the volume and speed of an individual players’ ball releases to provide a single score (See calculation 1).


$$\begin{array}{c} Ball\;release\;index=(Number\;of\;ball\;releases\;*\;Average\;ball\;release\;velocity)/100\\ \mathrm{The}\;\mathrm{calculation}\;\mathrm{of}\;a\;\mathrm{release}\;\mathrm{index}\;\mathrm{to}\;\mathrm{provide}\;a\;\mathrm{single}\;\mathrm{number}\;\mathrm{reflective}\;\mathrm{of}\;\mathrm{the}\;\mathrm{volume}\;\mathrm{and}\;\mathrm{speed}\;\mathrm{of}\;\mathrm{all}\;\mathrm{kicking}\;\mathrm{actions}. \end{array}$$ Calculation 1

### Statistical analysis

Having verified the assumption of normality using a Q-Q plot [24], general linear modelling was conducted within SPSS (v. 26; IBM, Chicago, USA) to establish estimated marginal mean values for the fixed variables of interest: ball releases (total, dominant leg, non-dominant leg), high-speed ball releases and ball release index (total, dominant leg, non-dominant leg). For each variable, variables included were the total output and the total output for the dominant and non-dominant kicking leg. Random variables (e.g., player age, calendar month) were screened for covariance [25], with Wald Z statistics (*p* > 0.05) indicating that no random intercept was required. In the event of a statistically significant *F* ratio, Sidak adjusted post-hoc pairwise comparisons between the estimated marginal means were analysed. Measures of effect size were calculated using partial eta-squared (η^2^) was computed to ascertain the magnitude of the number of games per week, within-microcycle, inter-positional and drill category differences, with the following descriptors attached: small (>0.02), medium (>0.13) and large (>0.26) [25]. Two-tailed statistical significance was accepted as p ≤ 0.05 [26].

## Results

### Fixture proximity

There were main effects of fixture proximity for both absolute (f = 32.71; p>0.000; η^2^–0.156) and relative (f = 19.85; p>0.000; η^2^–0.111) data in relation to ball releases, high-speed ball releases and ball release index (including dominant and non-dominant comparisons). Both absolute (Table 1) and relative (Table 2) data are presented below.

**Table 1:** Estimated marginal means (±SE) and 95% confidence interval values representative of the absolute frequency of ball releases, dominant ball releases, non-dominate ball releases, high-speed ball releases, ball release index, ball release index dominant and ball release index non-dominant for professional soccer players according to match proximity (match day [MD]).

	MD-4	MD-3	MD-2	MD-1	MD+1
**Releases (*f*)**	132.7 ± 39.1	139.8 ± 38.6	118.1 ± 29.4	82.7 ± 40.4	161.1 ± 51.2
	(126.6 to 138.8)	(134.6 to 145)	(114.4 to 121.9)	(79.8 to 85.7)	(155 to 167.2)
**Dominant releases (*f*)**	102.3 ± 33.9	109.1 ± 31.5	95.8 ± 26	65.9 ± 31.9	124 ± 40
	(97 to 107.6)	(104.9 to 113.4)	(92.5 to 99.1)	(63.5 to 68.2)	(119.3 to 128.8)
**Non-dominant releases (*f*)**	30.4 ± 18.7	30.7 ± 20.4	22.3 ± 12.1	16.9 ± 13.2	37.1 ± 19.4
	(27.5 to 33.3)	(27.9 to 33.4)	(20.8 to 23.9)	(15.9 to 17.8)	(34.8 to 39.4)
**High-speed releases (>15m/s) (*f*)**	13.4 ± 8.2	12.4 ± 8.6	16.8 ± 15	8.4 ± 10.1	17.6 ± 11.9
	(12.1 to 14.6)	(11.3 to 13.6)	(14.9 to 18.7)	(7.6 to 9.1)	(16.2 to 19)
**Release index**	128.6 ± 35.7	125.8 ± 38	107.3 ± 35.8	74 ± 40.3	145.5 ± 45.2
	(123 to 134.2)	(120.7 to 131)	(102.8 to 111.9)	(71 to 76.9)	(140.1 to 150.9)
**Release index dominant**	100.7 ± 33	99.9 ± 32.5	87.7 ± 31.2	59.2 ± 32.2	114.2 ± 36.1
	(95.6 to 105.9)	(95.5 to 104.3)	(83.8 to 91.7)	(56.8 to 61.5)	(110 to 118.5)
**Release index non-dominant**	27.8 ± 17.5	26.4 ± 17.7	19.6 ± 12.6	14.8 ± 13	31.4 ± 17.4
	(25.1 to 30.5)	(24.1 to 28.8)	(18 to 21.2)	(13.9 to 15.8)	(29.3 to 33.5)

MD-4- Match Day minus 4; MD-3 Match Day minus 3; MD-2- Match Day minus 2; MD-1- Match Day -1; MD+1- Match Day +1.

**Table 2:** Estimated marginal means (±SE) and 95% confidence interval values representative of the relative frequency of ball releases, dominant ball releases, non-dominate ball releases, high-speed ball releases, ball release index, ball release index dominant and ball release index non-dominant for professional soccer players according to match proximity (match day [MD]).

	MD-4	MD-3	MD-2	MD-1	MD+1
**Releases (*f*)**	1.2 ± 0.27	1.32 ± 0.42	1.9 ± 0.52	1.46 ± 0.57	2.01 ± 0.61
	(1.16 to 1.24)	(1.26 to 1.38)	(1.83 to 1.97)	(1.42 to 1.51)	(1.94 to 2.09)
**Dominant releases (*f*)**	0.93 ± 0.25	1.03 ± 0.3	1.55 ± 0.46	1.18 ± 0.45	1.56 ± 0.48
	(0.89 to 0.97)	(0.99 to 1.07)	(1.49 to 1.61)	(1.15 to 1.21)	(1.51 to 1.62)
**Non-dominant releases (*f*)**	0.27 ± 0.15	0.3 ± 0.23	0.36 ± 0.2	0.3 ± 0.21	0.47 ± 0.23
	(0.25 to 0.3)	(0.26 to 0.33)	(0.34 to 0.39)	(0.28 to 0.31)	(0.44 to 0.49)
**High-speed releases (>15m/s) (*f*)**	0.12 ± 0.07	0.11 ± 0.07	0.26 ± 0.2	0.14 ± 0.15	0.22 ± 0.15
	(0.11 to 0.13)	(0.1 to 0.12)	(0.23 to 0.28)	(0.13 to 0.15)	(0.2 to 0.24)
**Release index**	1.17 ± 0.23	1.18 ± 0.34	1.71 ± 0.52	1.3 ± 0.55	1.84 ± 0.56
	(1.13 to 1.2)	(1.13 to 1.22)	(1.65 to 1.78)	(1.26 to 1.34)	(1.77 to 1.9)
**Release index dominant**	0.92 ± 0.24	0.93 ± 0.28	1.4 ± 0.47	1.04 ± 0.44	1.44 ± 0.45
	(0.88 to 0.95)	(0.89 to 0.97)	(1.34 to 1.46)	(1.01 to 1.08)	(1.39 to 1.5)
**Release index non-dominant**	0.25 ± 0.14	0.25 ± 0.17	0.31 ± 0.19	0.26 ± 0.21	0.39 ± 0.21
	(0.23 to 0.27)	(0.23 to 0.27)	(0.29 to 0.33)	(0.24 to 0.27)	(0.37 to 0.42)

MD-4- Match Day minus 4; MD-3 Match Day minus 3; MD-2- Match Day minus 2; MD-1- Match Day -1; MD+1- Match Day +1.

### Playing position

**Fig 1:**
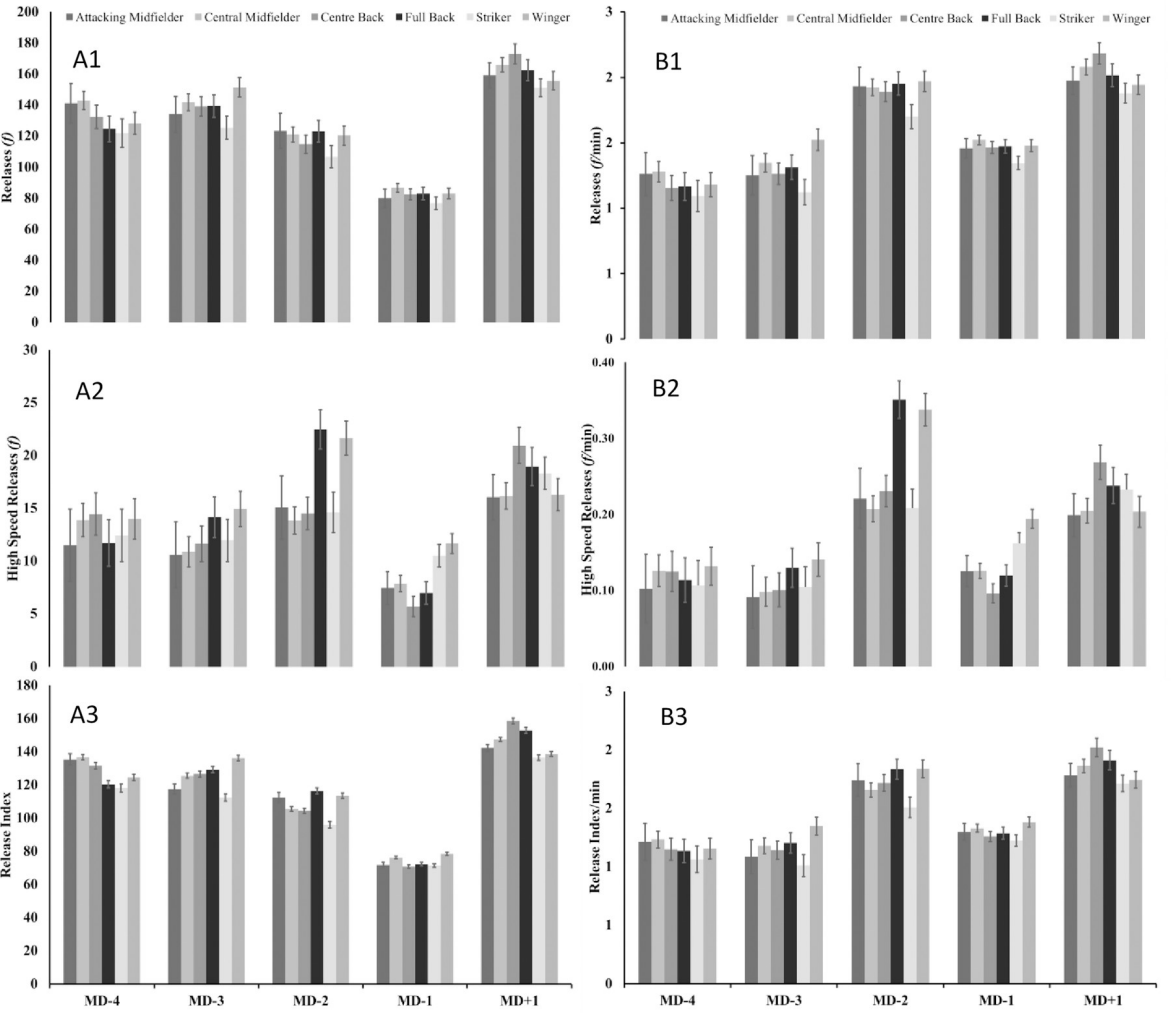
Absolute (A) and relative (B) estimated marginal means for number of ball releases (1), number of high-speed ball releases (2) and ball release index (3) during different training days according to match proximity (match day [MD]).

**Fig 2:**
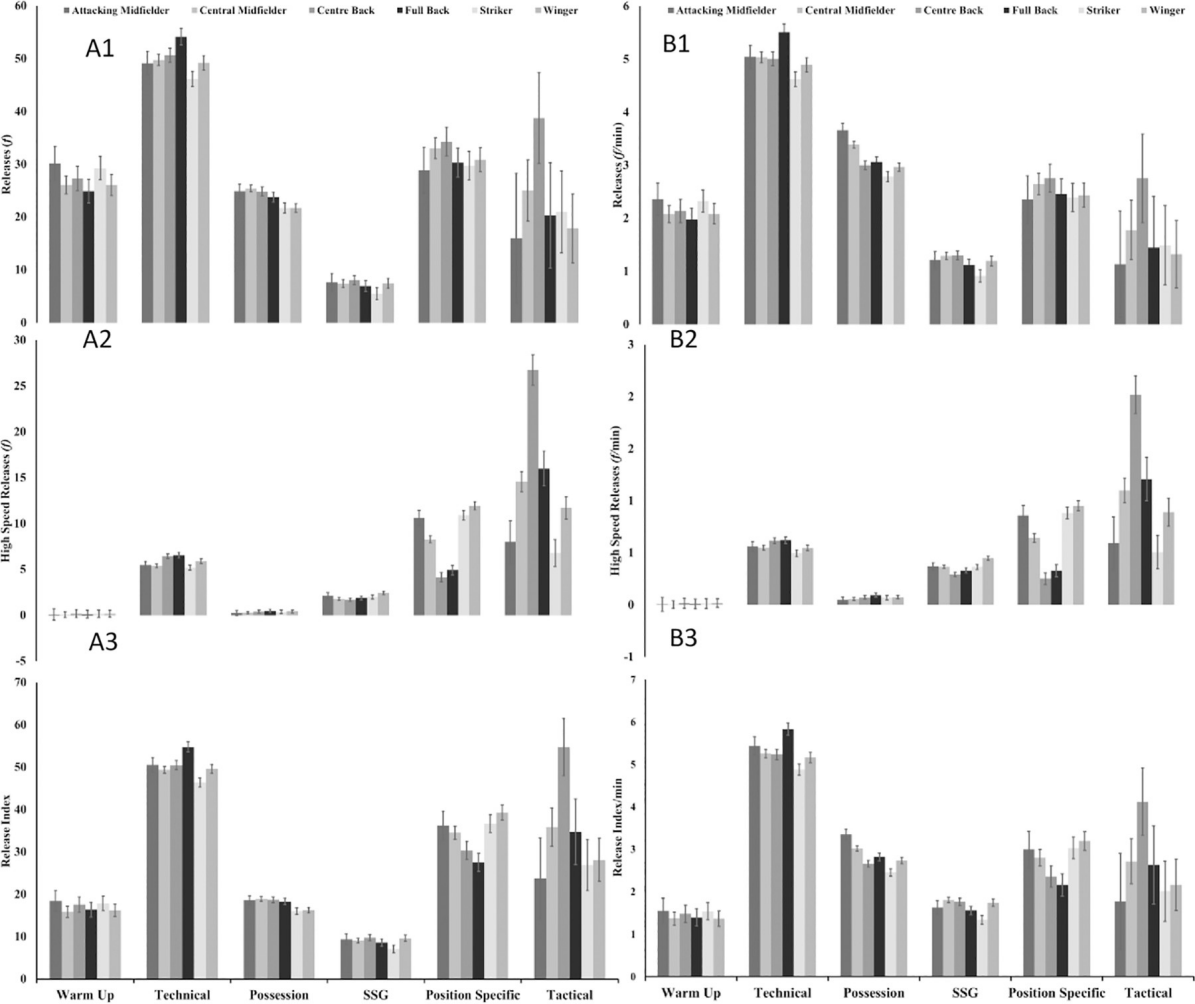
Absolute (a) and relative (b) estimated marginal means for number of ball releases (1), number of high-speed ball releases (2) and ball release index (3) during different training drills categories (warm-up, technical, possession, small-sided game [SSG], playing position specific and tactical) according to playing position.

Main effects of playing position were apparent for both absolute (f = 2.78; p>0.000; η^2^–0.016) and relative (f = 1.70; p>0.001; η^2^–0.011) data in relation to ball releases, high-speed ball releases and ball release index (including dominant and non-dominant comparisons; See Figs 1 and 2). Both wide defenders and wide midfielders had increased relative high-speed ball releases on a MD-2 (Fig 1), whereas centre backs had a higher amount of high-speed ball releases during tactical drills (Fig 2).

### Drill category

Drill category showed main effects for absolute (f = 231.55; p>0.000; η^2^–0.258) and relative (f = 156.99; p>0.000; η^2^–0.212) data in relation to ball releases, high-speed ball releases and ball release index (including dominant and non-dominant comparisons). SSG’s had the lowest total frequency and high-speed ball releases in comparison to any other drill for absolute and relative (Fig 2) outputs.

## Discussion

The aims of the current study were two-fold: i) examine the validity and reliability of high-speed kicking actions using foot-mounted IMU’s, ii) quantify soccer players within-microcycle, inter-positional differences in both the frequency and speed of technical actions. The main findings of the present study were: (i) foot-mounted IMU’s showed *good* concurrent validity and reliability for measuring ball release velocity during controlled passing actions; (ii) MD+1 had the highest absolute number of ball releases and ball release indexes compared to all other training days; (iii) A decrease in frequency of ball releases was observed as fixture proximity became closer to match-play; (vi) MD-2 and MD+1 had the highest number of absolute and relative high-speed ball releases in comparison to any other training day; (v) In comparison to other training drill categories, SSG’s had the lowest absolute and relative outputs for ball releases and ball release index, with both possessions and SSG’s showing the lowest high-speed ball releases across drill categories.

Quantifying soccer specific, non-locomotor activities is desirable for practitioners to integrate both physical and technical actions into player monitoring processes to assess all potential influences on soccer players performance and risk of injury [15, 23, 27]. In the current study, foot-mounted IMU’s showed *good* concurrent validity compared to high-speed cameras systems for quantifying lower-limb speed during kicking actions (r^2^- 0.96), with *moderate* to *large* reliability (3.9–14.35%). Insights into both the number and speed of technical actions using foot-mounted IMU’s can provide an automated, time-efficient alternative to manual coding, thus negating potential human measurement errors derived from false-positive and subjective interpretations of events [7, 28, 29], permitting enhanced accuracy for the quantification of players’ technical load. While the frequency of technical actions has previously been reported, showing *good* concurrent and inter-unit reliability against video analysis [7]. This is the first study to report the validity and reliability for kicking speeds within an applied environment using foot-mounted IMU technology. However, further research is required to explore our understanding of how performing different kicking speeds can impact both the physiological and mechanical costs associated with soccer training and match play [30], ultimately linking this with players performance or potential risk of injury [15].

**Fig 3:**
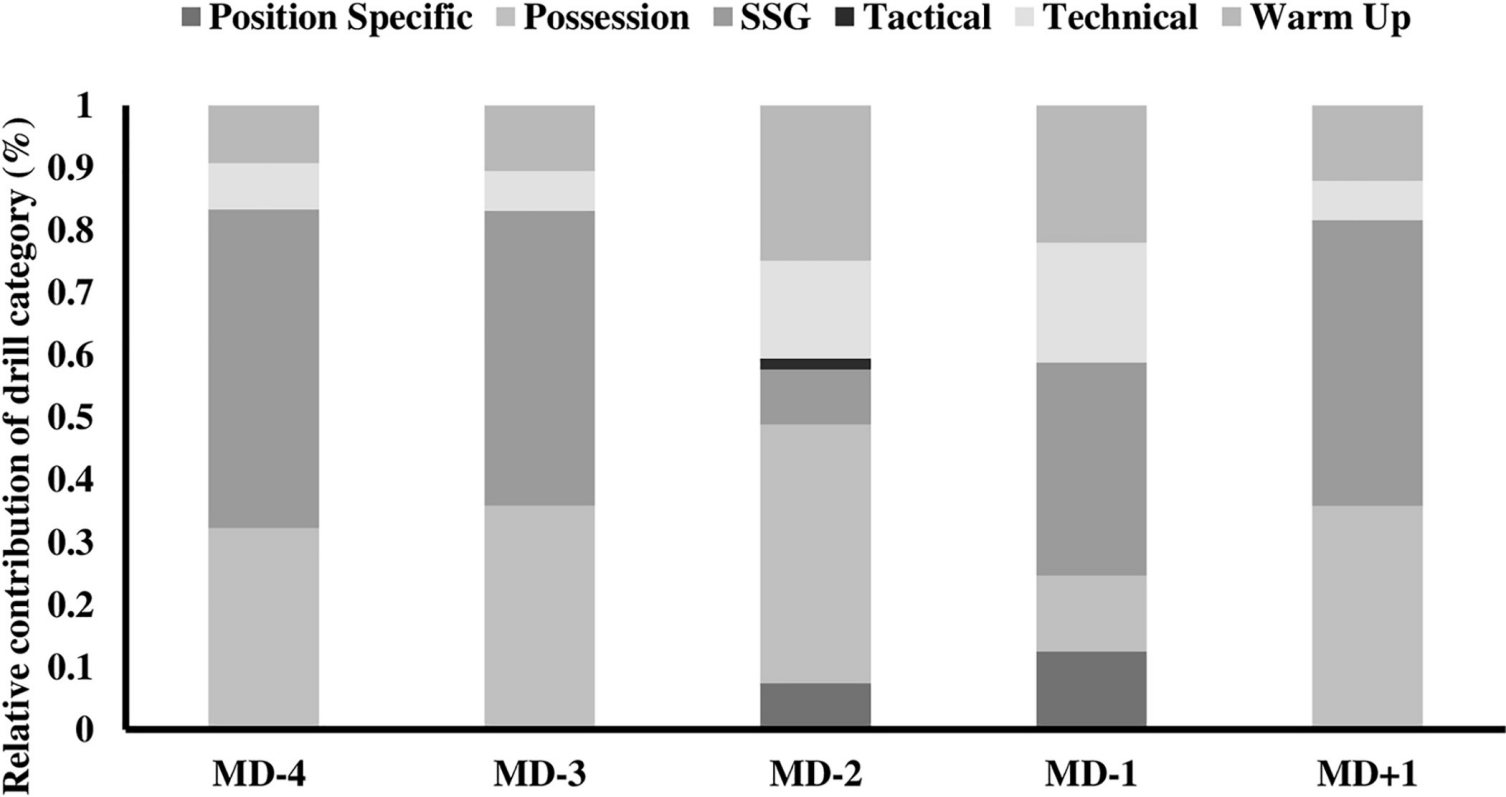
A percentage distribution of training drill categories (warm-up, technical, possession, small-sided game [SSG], playing position specific and tactical) utilised according to match proximity (match day [MD]).

Understanding the frequency of different player activities have underpinned the rationale behind training programmes in soccer, showing a physical taper within a microcycle towards match-day [4]. Despite this, the technical actions observed during soccer training, have been reported to follow a contradictory trend, with higher frequencies occurring on a MD-1 [7]. Within the current study, the frequency of technical actions was higher on days further away from match-play or immediately following a match, which compare to trends observed for external training load metrics [4]. Variation in drill types was seen on different training days, which may be associated with the differences in the frequency of the actions (Fig 3). Furthermore, MD+1 had the highest frequency and speed of technical actions performed, potentially as a coaching strategy to provide non-match-day starters (i.e., substitutes etc) exposure to technical actions they may have missed on a match-day [31]. Technical actions are negated during post-match conditioning for non-playing squad members, with MD+1 suggested as an alternative opportunity to expose non-playing squad members an opportunity to be ‘topped-up’ [31]. Furthermore, ball releases within the current study reported a larger frequency (0.9–31.2%) in comparison to previously reported data from a team in the same division (80.9 to 110.8 [7]). Differences within soccer teams training has been attributed to several factors (style of play, head coach, micro-cycle etc [2, 32]). The findings of this study, and those of previous studies [7] further identifies the requirement to explore multi-club studies, over a longer period of time to identify which contextual factors explain the variability within soccer training technical actions to better understand the complexities of accurately prescribing optimal technical outputs for players across the training week.

Understanding the positional demands within soccer has allowed practitioners to monitor and develop training sessions specific to the needs of that position [6, 33]. Positional differences were observed in the present study for high-speed kicking actions, specifically on a MD-2 for full-backs and wide players. This maybe a consequence of the increased exposure to technical drill categories on a MD-2. Within the current study, only drill category was considered for analysis as opposed to the individual specific drills. Anecdotally, coaches and support staff suggested this increased exposure to high-speed kicking actions, may have been associated with an individual drill for these positions (crossing and finishing), where the wide defenders and wide midfielders were required to perform multiple high-speed kicking actions in order to achieve passes over longer distances which likely occur during full match-play. Such actions have been associated with goal scoring opportunities within match play, which may suggest the coaches use of them within their training programme to support players learning objectives [34]. However, the increased strain on players performing high-speed kicking actions repetitively has been shown to elicit an increased physiological strain on players [15, 16], which in turn has been associated with the increased incidence of injury [10, 35, 36].

Kicking actions have been associated with potential risk of injury during soccer activities [10, 37], with limited evidence supporting the frequency or intensities of these actions in training [7]. The ability to monitor and understand the implications of kicking speed and the physical requirements of kicking during soccer specific activities may help inform practitioners training and return to training prescription [35, 37]. Within the current study, technical, tactical, and position-specific drills had the highest high-speed ball release frequencies across all positions in comparison to other training drill types (warm-ups, SSG’s and Possessions; Fig 2). However, observations made using the proposed ball release index in the current study, identified that only SSG’s showed a reduced technical output compared to other drills, suggesting a higher frequency of technical actions were performed within possession and warm-up drills compared to SSG’s (Fig 2). It is likely that this low technical output is explained by coaches in the present study electing to utilise SSG’s as a training tool to simulate locomotive elements of match-play, that elicit similar, if not elevated physiological demands in comparison to full match-play [2, 32, 38]. Given the low-frequency and speed of technical actions in the current study, the use of SSG’s may not reflect the absolute or relative technical demands of match-play as previously suggested [7, 32]. However, SSG’s represent one of the most utilised training types during the current study (Fig 3) and previous soccer training [2, 4, 32]. Decision making skills under game-like scenarios are key to challenge players within a training environment for coaches, which may suggest why SSG’s are a popular training tool in elite and academy soccer [34, 39] and assessing all forms of decision making is beyond the scope of the current study’s findings. Despite the potential benefits of SSG’s, the low technical outputs reported could have possible implications if players are not conditioned towards the technical intensities of match-play. This may result in reduced technical performance or increased risk of injury, and it is therefore important that coaches consider the prescription of other drill types with higher relative technical outputs in addition to SSG’s if they intend to expose players to higher volumes and intensities of technical load. Further exploration is warranted to link the physical, technical, and cognitive skills associated within SSG’s to understand why they are used, how best to monitor players responses to them and how they may be adapted to increase the technical actions observed.

The current study has some limitations. Findings of the study demonstrate evidence that tapering of technical actions can be achieved within a professional training regime, however, the study only looks at kicking load in isolation. Further research is required, integrating locomotive GPS metrics and foot-mounted IMU technical actions to give a broader representation of external training load and to confirm whether a true physical taper can be prescribed concurrently. In addition to the taper of technical actions observed, the study identifies drill types that expose players to differing frequencies and intensities of technical outputs, although there is no evidence in the literature to suggest optimal levels for performance or injury prevention. The championship-winning success of the team suggests that the volumes and intensities prescribed were effective for performance, however, further multi-club research exploring the link of high-speed kicking actions to injury within training and match play is required [15, 35] which could also help to inform return to play protocols and identify key objective markers related to technical actions. It was also beyond the scope of the present study to make comparisons between different types of drill within each individual category. Further research, comparing effect of player number, pitch size, playing duration, and rules and restrictions within the drill on technical output is required, in order to provide practitioners with further detail on manipulating individual drills to meet performance or physical objectives.

The present study provides novel information regarding the frequency and intensity of technical actions of professional soccer players, which may be relevant to practitioners in the prescription of external load through soccer training programmes. Foot-mounted IMU’s are seen to show *good* concurrent validity and reliability for measuring ball release velocity during controlled passing actions. Findings of the study suggest that a taper in frequency of technical actions in addition to the traditional taper of locomotive activities [4] in sessions close in proximity to a fixture is possible, and that MD+1 sessions expose non-playing players to high frequency and intensity of absolute and relative technical actions. Technical, tactical, and position-specific drills have the highest high-speed ball release frequencies across all positions in comparison to other training drill types, with SSG’s producing the lowest technical output. Insights into absolute and relative frequency and intensity of releases in different drill types, provide practitioners with valuable information on technical outputs that can be manipulated through drill selection and duration, during the process of planning training programmes to produce the desired outcomes to compliment already established locomotive objectives.

## Supporting information

S1 Table The six categories of training drill utilised in the training programme, with corresponding operational definitions [2]. (DOCX)
