# Supplementary material for: Quantifying volume and high-speed technical actions of professional soccer players using foot-mounted inertial measurement units
Source: PLoS One. 2022 Feb 3;17(2):e0263518. doi: 10.1371/journal.pone.0263518 (PMC8812977; doi:10.1371/journal.pone.0263518)
Supplement: S1 Table — (DOCX) [file pone.0263518.s001.docx]

**Supplementary Table**

Supplementary table 1. The six categories of training drill utilised in the training programme, with corresponding operational definitions [2].

| **Drill Category** | **Operational Definition** |
| --- | --- |
| **Position specific** | *Drills aimed at specific units of the team (i.e., defenders, midfielders and strikers), with players separated according to their position, and coached as a unit or an individual.* |
| **Possession** | *Drills designed to mimic similar demands of match play, with the aim being to keep the ball away from the opposing team, with no goals to score in.* |
| **Small-Sided Games** | *Drills expected to replicate the demands of match play, with a reduced number of players, a reduced pitch size, and specific rules to elicit the desired intensity, with goals to score in.* |
| **Tactical** | *Drills intended to educate players as to the tactical roles they occupy within the team formation, inclusive of open play and set-piece exercises.* |
| **Technical** | *Drills designed to work on a soccer-specific skill (e.g., dribbling, passing, crossing, shooting).* |
| **Warm-Up** | *Drills intended to prepare the players, both physically and technically, for the forthcoming training session.* |
